# Risk factors for failure of distal femoral nonunion repair

**DOI:** 10.1007/s00590-025-04460-9

**Published:** 2025-08-10

**Authors:** Robert K. Wagner, Jochem H. Raats, Noa H. M. Ponds, Jacob S. Borgida, Devon T. Brameier, Mitchel B. Harris, Peter Kloen, Stein J. Janssen, Thuan V. Ly, Michael J. Weaver

**Affiliations:** 1 Department of Orthopedic Surgery and Sports Medicine, Amsterdam UMC, Amsterdam, The Netherlands; 2 Department of Orthopaedic Surgery, Brigham and Women’s Hospital, Boston, United States; 3 Department of Orthopaedic Surgery, Massachusetts General Hospital, Boston, United States; 4 Musculoskeletal Health, Amsterdam Movement Sciences, Amsterdam, The Netherlands; 5 Harvard Medical School Orthopaedic Trauma Initative, Boston, United States

**Keywords:** Distal, Supracondylar, Femur, Nonunion, Repair, Revision

## Abstract

**Introduction:**

There is limited evidence to guide treatment strategies for native and periprosthetic distal femoral nonunions. The aim was to determine factors associated with failure of distal femoral nonunion repair.

**Methods:**

All adult patients undergoing operative repair for a distal femoral nonunion from 2004 to 2023 at two Level 1 Trauma Centers with ≥ 6 months follow-up were identified. The primary outcome was additional nonunion surgery. Univariate logistic regression was performed to determine associations of patient, initial fracture, nonunion, and treatment characteristics with additional nonunion surgery.

**Results:**

Eighty-six patients (median age 63 years, 63% female) were included. Definitive fixation was most often a non-augmented lateral locking plate (LLP, 52%), 95-degree-blade-plate (BP, 29%), or augmented LLP (15%). Augmented fixation was defined as the addition of a medial or endosteal plate or intramedullary nail. Fourteen patients (16%) required additional nonunion surgery. In univariate logistic regression analysis, initial high-energy injuries (OR: 4.18, p = 0.044), increasing number of previous surgeries (OR: 1.94, p = 0.007), and treatment with retention of previous implants (OR: 5.25, p = 0.010) or bone morphogenetic protein use (OR: 5.82, p = 0.005) were associated with increased odds of additional nonunion surgery; whereas treatment with BP constructs (vs. non-augmented LLPs, OR: 0.11, p = 0.044) reduced odds. Rates of additional nonunion surgery were 12/45 (27%) for non-augmented LLP, 1/13 (7.7%) for augmented LLP, and 1/25 (4.0%) for BP constructs. When excluding patients treated with retention of previous implants, rates were 7/35 (20%) for non-augmented LLP, 0/8 (0%) for augmented LLP, and 1/25 (4%) for BP constructs. There were differences across constructs, including for rates of initial intra-articular fractures (49% vs. 0% vs. 13%), and use of autograft (51% vs. 62% vs. 16%) and bone morphogenetic protein (44% vs. 31% vs. 8.0%).

**Conclusions:**

Approximately 1 in 6 patients required additional nonunion surgery. In unadjusted analyses, initial high-energy injuries and more prior surgeries were associated with increased odds for additional nonunion surgery, suggesting that the severity of the initial injury is associated with subsequent nonunion treatment outcomes. The current study findings suggest that distal femoral nonunion repair should be based on revision fixation using augmented lateral locking plate (dual-plate or nail-plate) or blade plate constructs. However, these findings are based on unadjusted comparisons. Larger studies with sufficient power to correct or stratify for confounding are needed to further define optimal treatment.

## Introduction

Despite advances in implant design and surgical technique in the treatment of distal femur fractures, there is still a high risk of nonunion, ranging from 6 to 13% when initially treated with lateral locking plates [1–5]. The mainstay treatment is surgical repair to restore the mechano-biological environment of the nonunion, which can be challenging due to poor bone stock, bone loss, disuse osteopenia, poor vascularization, soft tissue scarring, stiffness, muscle atrophy, (subclinical) infection, or presence of (failed) implants.

Treatment principles include (staged) debridement and restoration of alignment followed by stable fixation, ideally combined with compression across the nonunion site, allowing for early motion. Fixation constructs typically include the use of lateral locked plates, blade plates, or intramedullary nails which may also be combined in augmented fixation constructs (e.g., nail-plate or dual-plate combinations). These can be supplemented with bone graft, bone substitutes, or local antimicrobial treatment in the presence of infection. Soft tissue releases such as Judet’s quadriceps plasty may be needed to help increase knee motion. Despite the relative frequency of reports on distal femoral nonunion in the literature, there remains limited evidence to help guide treatment strategies [6–10]. Previous studies are limited by small sample size and often report on a single technique, which is problematic for generalizability and identification of factors to optimize treatment. Specifically, there is limited comparative data for common fixation constructs used in nonunion repair.

The primary objective of this study was to determine the rate of additional nonunion surgery after operative treatment for a distal femoral nonunion (including conversion to distal femoral replacement or above-knee-amputation). The secondary objective was to identify factors that were associated with additional nonunion surgery, and specifically the association with repair with (1) non-augmented lateral locking plate constructs, (2) augmented lateral locking plate constructs, and (3) augmented or non-augmented 95-degree-blade-plate constructs.

## Methods

After Institutional Review Board approval (protocol number: 2024P000624) all consecutive adult patients who underwent operative treatment for a distal femoral nonunion between 2004 and 2023 were identified at two Level 1 Trauma and Tertiary Referral centers. Patients were retrieved from an institutional research patient data registry using CPT codes 27450, 27470, 2772, and ICD-10 codes for malunion, delayed union, and nonunion after femur fractures (S72).

Distal femoral nonunions were defined as AO/OTA 33A or 33C fractures without progression of healing on sequential radiographic follow-up or mechanical implant failure, and that were not expected to heal without further surgery as determined by the treating surgeon. Patients with nonunions from a pathological fracture or those with less than 6 months of clinical or radiographic follow-up were excluded. Planned bone grafting procedures in the setting of staged management for open distal femur fractures with bone loss were not considered nonunion procedures. The first nonunion surgery for single-stage treatment or the definitive nonunion surgery for staged nonunion treatment at the authors’ institutions was considered the index nonunion surgery. Patients who had undergone previous nonunion surgeries at an outside institution were included. Overall 17 orthopaedic surgeons (13 orthopaedic trauma fellowship trained) were involved in treatment.

### Outcomes

The primary outcome variable was additional nonunion surgery for persistent nonunion after the index nonunion surgery. Additional nonunion surgery was defined as any surgical procedure after the index surgery with revision of the fixation or (staged) bone grafting for persistent nonunion or conversion to a distal femoral replacement or above-knee amputation. Patients who did not require additional nonunion surgery within the 6-month follow-up were considered healed. Treatment of fracture-related infection (FRI) after the index nonunion surgery with irrigation and debridement without revision of fixation or bone grafting was not considered an additional nonunion surgery.

### Explanatory variables

Baseline patient, initial fracture, and nonunion characteristics included age, sex, diabetes mellitus, active tobacco use, body mass index (BMI), American Society of Anesthesiologists (ASA) physical status, initial periprosthetic fracture (above total knee arthroplasty [TKA]), AO/OTA fracture classification[11], initial injury mechanism (high vs. low energy), open fracture status, number of previous surgeries, previous surgeries for nonunion at an outside institution, Weber and Çech classification (oligotrophic/atrophic vs. hypertrophic), type of implant present at presentation, mechanical implant failure, and diagnosis of FRI. Mechanical implant failure was defined as the bending or breaking of plates or nails, screw cut-out, or failure of the screw-plate interface. FRI was determined according to the FRI consensus criteria based on documentation of preoperative and intraoperative clinical assessment or intraoperative culture samples [12]. There was heterogeneity in culture protocols used, with 85% of nonunions being cultured with tissue or hardware samples or swabs, with a median of 1 (interquartile range [IQR: 1–2]) culture per nonunion.

**Fig. 1:**
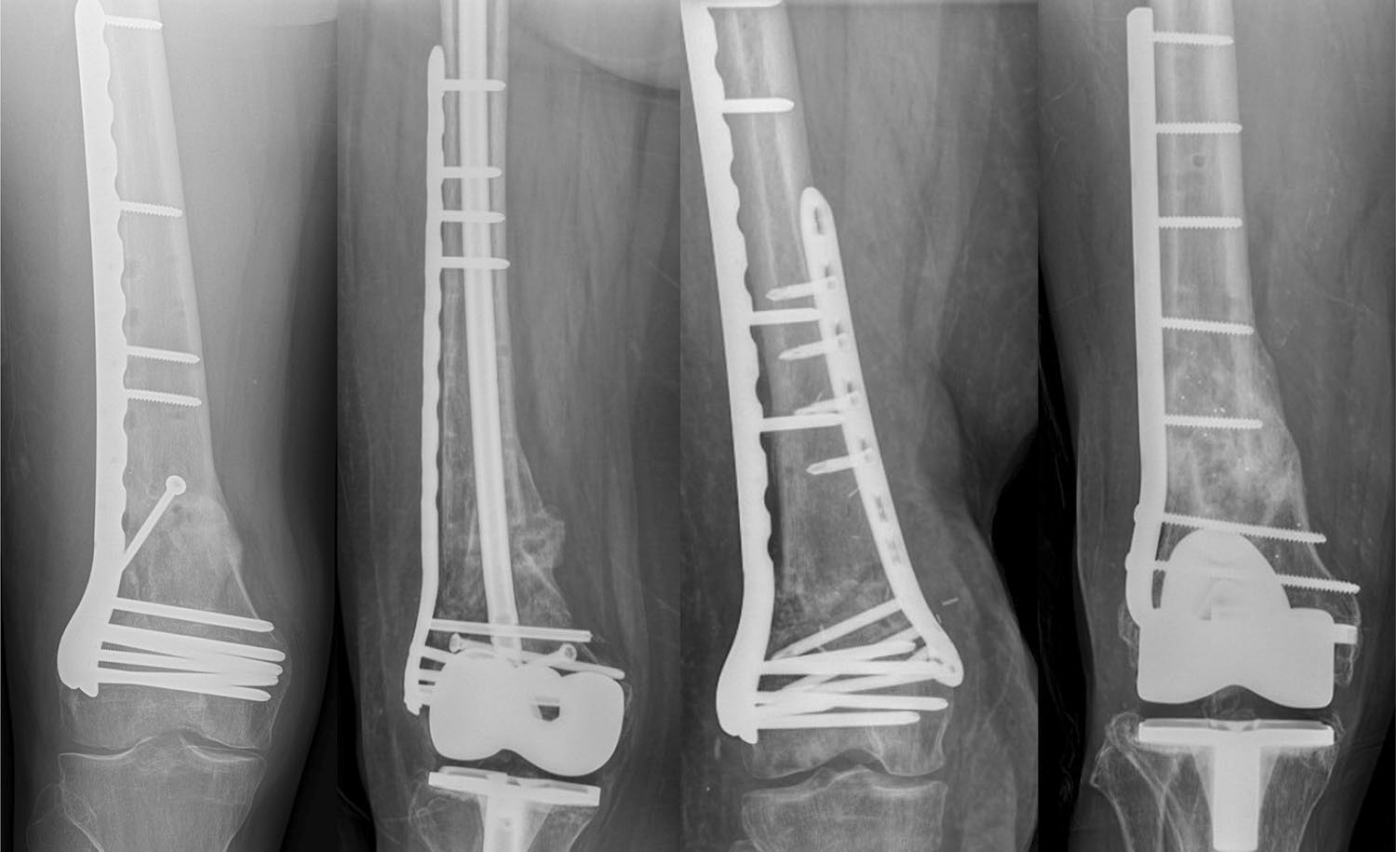
Fixation constructs. From left to right: non-augmented lateral locking plate; augmented lateral locking plate with intramedullary nail; augmented lateral locking plate with medial plate; non-augmented blade plate

Treatment characteristics included number of treatment stages, retention of previous implants, definitive fixation construct, and use of autograft (iliac crest bone graft [ICBG], Reamer-Irrigator-Aspirator [RIA]), or bone graft substitutes (demineralized bone matrix [DBM], cancellous chips or bone morphogenetic protein [BMP]). For comparison, fixation constructs were stratified in non-augmented lateral locking plate vs. augmented lateral locking plate vs. augmented or non-augmented blade plate constructs (Fig. 1). Augmented fixation was defined as the addition of a medial or endosteal plate or intramedullary nail.

### Statistical analysis

No ante-hoc sample size calculation was performed and all consecutive cases that met eligibility criteria were included. Continuous variables were summarized as medians with IQR, and categorical variables were summarized using frequencies with percentages. Differences in initial fracture, nonunion, and treatment characteristics between fixation constructs were assessed using Kruskal-Wallis rank sum test for continuous, or Pearson’s Chi-squared test or Fisher’s exact for categorical variables. To evaluate potential associations between explanatory variables (patient, initial fracture, nonunion, and treatment characteristics) and additional nonunion surgery, univariate logistic regression was used to estimate odds ratios (ORs) with corresponding 95% confidence intervals (CIs). Given the low event rate (n = 14), multivariable analysis was not performed. To aid clinical interpretability, 95% CIs were determined for overall rates of additional nonunion surgery and for fixation constructs, stratified by retention of implants. Given the low number of patients treated with intramedullary nailing alone (n = 3), these patients were not included in the fixation construct comparison. Significance was set at a two-tailed alpha of 0.05 without adjustment for multiple testing, given that these assessments were exploratory. All statistical tests were performed using R (R: A Language and Environment for Statistical Computing; R Foundation for Statistical Computing; 2023, Vienna, Austria).

## Results

*Descriptive data:* Eighty-six patients with 86 nonunions met the inclusion criteria. The median age was 63 years (IQR: 56–75), 54 (63%) were female, and 14 (16%) were active tobacco users at the time of the index nonunion repair (5 patients with unknown tobacco use, Table 1). Twenty patients (23%) had a nonunion above a TKA and 19 (22%) initially had an open fracture. Nine (10%) patients had undergone at least one prior nonunion surgery at an outside institution before the index nonunion repair at the authors’ institution and the median time from the initial injury was 9 months (IQR: 6–15). The most common implant at presentation (i.e., before the index surgery) was a single lateral plate (n = 67, 78%). Thirty-seven patients (45%) presented with mechanical implant failure. Ten patients (11%) were diagnosed with an infected nonunion during the treatment course based on either preoperative or intraoperative clinical findings, or intraoperative cultures. Eight (9.3%) patients underwent staged treatment for either infection or presumed infection (n = 7) or a bone defect (n = 1). Fifteen patients were treated with retention of previous implants. The most common definitive fixation constructs were based on lateral locking plates (n = 58, 67%) or blade plates (n = 25, 29%) (Table 2). These were augmented (i.e., addition of a medial plate, endosteal plate, or intramedullary nail) in 22% of the lateral locking plate and 24% of the blade plate constructs**.**

**Table 1:** Baseline patient, initial fracture, and nonunion characteristics

	All	No additional nonunion surgery	Additional nonunion surgery	OR (95% CI)	p-value
	(n = 86)	(n = 72)	(n = 14)		
**Patient characteristics**					
Age, years, median (IQR), 10-year increase	63 (56–75)	67 (56–78)	58 (52–61)	0.73 (0.52–1.02)	0.067
Female	54 (63%)	48 (67%)	6 (43%)	0.38 (0.11–1.20)	0.099
Diabetes Mellitus	21 (24%)	16 (22%)	5 (36%)	1.94 (0.53–6.50)	0.288
Tobacco use	14 (16%)	13 (18%)	1 (7.1%)	0.32 (0.02–1.84)	0.292
BMI, kg/m^2^, median (IQR), 5-point increase	30 (25–35)	28 (24–35)	33 (31–40)	1.38 (0.91–2.07)	0.112
ASA Classification					
I-II	52 (60%)	43 (60%)	9 (64%)	*Reference*	
III-IV	34 (40%)	29 (40%)	5 (36%)	0.82 (0.23–2.64)	0.750
**Initial fracture characteristics**					
Initial high-energy injury mechanism	37 (47%)	28 (42%)	9 (75%)	4.18 (1.13–20.1)	**0.044**
Periprosthetic fracture	20 (23%)	16 (22%)	4 (29%)	1.40 (0.35–4.83)	0.608
Intra-articular fracture	25 (29%)	18 (25%)	7 (50%)	2.94 (0.90–9.76)	0.072
Open fracture	19 (23%)	14 (20%)	5 (38%)	2.54 (0.68–8.90)	0.147
**Nonunion characteristics**					
Total number of previous surgeries, median (IQR), 1-unit increase	1.00 (1.00–1.00)	1.00 (1.00–1.00)	2.00 (1.00–3.50)	1.94 (1.28–3.34)	**0.007**
Previous nonunion surgery	9 (10%)	6 (8.3%)	3 (21%)	3.00 (0.57–13.3)	0.158
Nonunion duration in months, median (IQR), 1-month increase	9 (6–15)	10 (7–16)	7 (6–10)	0.95 (0.85–1.01)	0.213
Atrophic/oligotrophic nonunion	76 (89%)	64 (90%)	12 (86%)	0.66 (0.14–4.76)	0.625
Implant at presentation					
Single lateral plate	67 (78%)	58 (81%)	9 (64%)	*Reference*	
Intramedullary nail	8 (9.3%)	6 (8.3%)	2 (14%)	2.15 (0.29–11.2)	0.391
Nail-plate	3 (3.5%)	2 (2.8%)	1 (7.1%)	3.22 (0.14–37.2)	0.359
Other	8 (9.3%)	6 (8.3%)	2 (14%)	2.15 (0.29–11.2)	0.391
Mechanical implant failure	37 (45%)	31 (46%)	6 (43%)	0.90 (0.27–2.85)	0.852
Infected nonunion	10 (12%)	8 (11%)	2 (14%)	1.15 (0.43–2.49)	0.735

Bold indicates statistical significance (*p* < 0.05)

Missing values for: tobacco use (n = 5, 5.8%), BMI (n = 22, 26%), initial high-energy injury mechanism (n = 7, 8.1%), intra-articular fracture (n = 1, 1.2%), open fracture (n = 2, 2.3%), atrophic/oligotrophic nonunion (n = 1, 1.2%), mechanical implant failure (n = 4, 4.7%). Abbreviations: *BMI* body mass index, *ASA* American Society of Anesthesiologists Physical Status, *IQR* interquartile range, *OR* Odds Ratio, *CI* Confidence Interval

**Table 2:** Treatment characteristics

	All	No additional nonunion surgery	Additional nonunion surgery	OR (95% CI)	p-value
	(n = 86)	(n = 72)	(n = 14)		
**Treatment characteristics**					
Fixation construct					
Non-augmented lateral locking plate	45 (54%)	33 (48%)	12 (86%)	*Reference*	
Augmented lateral locking plate	13 (16%)	12 (17%)	1 (7.1%)	0.23 (0.01–1.36)	0.178
Blade plate	25 (30%)	24 (35%)	1 (7.1%)	0.11 (0.01–0.64)	**0.044**
Other	3 (3.5%)	3 (4.2%)	0 (0%)	-	-
Retention of implants	15 (17%)	9 (13%)	6 (43%)	5.25 (1.45–19.0)	**0.010**
Autograft (RIA, ICBG, or local callus)	36 (42%)	27 (38%)	9 (64%)	3.00 (0.94–10.6)	0.071
Bone graft substitute	28 (33%)	22 (31%)	6 (43%)	1.70 (0.51–5.50)	0.372
BMP	26 (30%)	17 (24%)	9 (64%)	5.82 (1.77–21.3)	**0.005**
DBM	16 (19%)	11 (15%)	5 (36%)	3.08 (0.82–10.8)	0.082
Cancellous chips	8 (9.3%)	7 (9.7%)	1 (7.1%)	0.71 (0.04–4.52)	0.762

Bold indicates statistical significance (*p* < 0.05)

Abbreviations: *ICBG* iliac crest bone graft, *RIA* Reamer-Irrigator-Aspirator, *BMP* bone morphogenetic protein, *DBM* demineralized bone matrix, *IQR* interquartile range, *OR* Odds Ratio, *CI* Confidence Interval

*Rate of additional surgery for persistent nonunion*: Of all patients, 72 (84% [95% CI: 74% to 90%]) successfully healed their nonunion while 14 (16% [95% CI: 9.5% to 26%]) required additional nonunion surgery after their index surgery. Of these 14 patients, 2 ultimately underwent above-knee-amputation and 2 underwent distal femoral replacement. One additional patient developed a FRI after the index nonunion surgery, which was treated with two irrigation and debridement procedures and placement of antibiotic beads, without requiring revision fixation or additional bone grafting. Characteristics of the 14 patients who had an additional nonunion surgery or were converted to above-knee-amputation or distal femoral replacement are detailed in Table 3.

**Table 3:** Patients undergoing additional nonunion surgery

Patient and nonunion characteristics								Index nonunion surgery characteristics				
**Age/Sex**	**Periprosthetic**	**Initial open fracture**	**Number of previous nonunion surgeries**	**Nonunion duration (months)**	**Weber and Çech**	**Implant at presentation**	**Mechanical failure**	**Infected nonunion**	**Stages**	**Definitive fixation construct**	**Retention of previous implant**	**Graft and substitutes**
59M	No	Closed	0	4.8	Oligotrophic/atrophic	Single lateral plate	No	No	Single	Non-augmented LLP	Yes	RIA + Cancellous chips
62M	No	Open	0	5.5	Oligotrophic/atrophic	Single lateral plate	Yes	No	Single	Non-augmented LLP	No	ICBG + DBM + BMP
24M	No	Open	0	7.3	Oligotrophic/atrophic	Single lateral plate	No	No	Two-stage	Non-augmented LLP	Yes	ICBG
72F	Yes	Closed	0	6.4	Oligotrophic/atrophic	Single lateral plate	Yes	No	Single	Non-augmented LLP	No	BMP
70F	Yes	Closed	1	27.0	Oligotrophic/atrophic	Lateral plate + IMN	Yes	No	Single	LLP + IMN	Yes	ICBG + BMP
74M	Yes	Closed	2	6.1	Oligotrophic/atrophic	BP	No	No	Single	BP	No	None
51M	No	Closed	0	3.7	Oligotrophic/atrophic	IMN	No	No	Single	Non-augmented LLP	No	BMP
58F	No	Open	0	14.9	Oligotrophic/atrophic	Single lateral plate	Yes	No	Single	Non-augmented LLP	Yes	ICBG + DBM + BMP
57M	No	Closed	0	7.4	Hypertrophic	Single lateral plate	Yes	No	Single	Non-augmented LLP	No	DBM + BMP
36M	No	Open	0	14.1	Oligotrophic/atrophic	Single lateral plate	No	Yes	Single	Non-augmented LLP	No	ICBG
53F	No	Open	0	4.7	Oligotrophic/atrophic	Single lateral plate	No	No	Single	Non-augmented LLP	Yes	ICBG + BMP
57F	No	Closed	0	6.9	Oligotrophic/atrophic	IMN	Yes	Yes	Two-stage	Non-augmented LLP	No	ICBG + DBM + BMP
41M	No	missing	4	9.5	Oligotrophic/atrophic	External fixator	No	Yes	Single	Non-augmented LLP	No	DBM + BMP + Fibula
59F	Yes	Closed	0	10.5	Hypertrophic	Single lateral plate	No	No	Single	Non-augmented LLP	Yes	ICBG

Additional nonunion surgery characteristics						
**Age/Sex**	**Additional nonunion surgery 1 indication**	**Additional nonunion surgery 1 type**	**Additional nonunion surgery 2 indication**	**Additional nonunion surgery 2 type**	**Additional nonunion surgery 3 indication**	**Additional nonunion surgery 3 type**
59M	Persistent nonunion	ROH + BP under compression + ICBG				
62M	Persistent nonunion	ROH + LLP + ICBG + BMP (OP-1)	Persistent nonunion	I&D + vascularized fibula + OP-1		
24M	Persistent nonunion	I&D + BMP + cancellous chips	Bone defect	ROH + BMP		
72F	Persistent nonunion	Planned stage 1: ROH + cement spacer		Planned stage 2: DFR		
70F	Persistent nonunion + mechanical failure	ROH + LLP + decortication + ICBG + DBM				
74M	Persistent nonunion	ROH + BP + decortication				
51M	Persistent nonunion + mechanical failure	ROH + LLP + fibula strut + DBM	Persistent nonunion + mechanical failure	ROH + BP under compression + DBX		
58F	Persistent nonunion + mechanical failure	ROH + DCS + ICBG				
57M	Persistent nonunion + mechanical failure	ROH + BP + ICBG + BMP (OP-1)	Persistent nonunion + mechanical failure	ROH + BP under compression + RIA		
36M	Persistent nonunion + FRI	AKA				
53F	Persistent nonunion	AKA	Wound problems	Split skin graft	Poor prosthesis fit	Revision AKA
57F	Persistent nonunion + mechanical failure	ROH + I&D + antibiotic coated IMN	Persistent nonunion	DFR		
41M	Persistent nonunion + mechanical failure	ROH + LLP + DBM	Persistent nonunion + mechanical failure	ROH + LLP + Cancellous chips + BMP		
59F	Persistent nonunion	ROH + decortication + ICBG				

Abbreviations: *BP* blade plate, *IMN* intramedullary nail, *LLP* lateral locking plate, *FRI* fracture-related infection, *RIA* Reamer-Irrigator-Aspirator, *ICBG* iliac crest bone graft, *DBM* demineralized bone matrix, *BMP* bone morphogenetic protein, *I&D* irrigation and debridement, *ROH* removal of hardware, *DFR* distal femoral replacement, *AKA* above-knee amputation, *OP-1* Osteogenic Protein 1

*Risk factors for additional surgery for persistent nonunion:* In univariate logistic regression analysis, initial high-energy injuries (OR: 4.18, p = 0.044), increasing number of previous surgeries (OR: 1.94, p = 0.007), treatment with retention of previous implants (OR: 5.25, p = 0.010), and treatment with BMP (OR: 5.82, p = 0.005) were associated with increased odds of additional nonunion surgery (Tables 1 and 2). There was a nonsignificant association between increasing age (OR: 0.73, p = 0.067), intra-articular fractures (OR: 2.94, p = 0.072), and autograft (OR: 3.00, p = 0.071) or DBM use (OR: 3.08, p = 0.082) with additional nonunion surgery.

*Comparison of fixation constructs:* Differences between nonunion and treatment characteristics across the three fixation construct groups are displayed in Table 4. Treatment with blade plate constructs reduced odds of additional nonunion surgery (vs. non-augmented lateral locking plates, OR: 0.11, p = 0.044), while treatment with augmented lateral locking plates did not show a significant association (OR: 0.23, p = 0.178) (Table 2^2^. The rate of additional nonunion surgery was 12/45 (27%) for non-augmented lateral locking plates, 1/13 (7.7%) for augmented lateral locking plates, and 1/25 (4.0%) for blade plate constructs (Table 5). When excluding patients treated with retention of previous implants, rates were 7/35 (20%) for non-augmented lateral locking plates, 0/8 (0%) for augmented lateral locking plates, and 1/25 (4%) for blade plate constructs.

**Table 4:** Comparison of initial fracture, nonunion, and treatment characteristics by fixation construct category

	**Non-augmented lateral locking plate**	**Augmented lateral locking plate (n = 13)**	**Augmented and non-augmented blade plate**	**p-value**
	**(n = 45)**		**(n = 25)**	
**Initial fracture characteristics**				
Initial high-energy injury mechanism	25 (60%)	3 (27%)	9 (39%)	0.089
Periprosthetic fracture	7 (16%)	7 (54%)	6 (24%)	**0.020**
Intra-articular fracture	22 (49%)	0 (0%)	3 (13%)	** < 0.001**
Open fracture	13 (30%)	2 (15%)	4 (17%)	0.481
**Nonunion characteristics**				
Total number of previous surgeries, median (IQR)	1.00 (1.00—1.00)	1.00 (1.00—1.00)	1.00 (1.00—2.00)	0.914
Previous nonunion surgery	1 (2.2%)	2 (15%)	5 (20%)	**0.024**
Nonunion duration in months, median (IQR)	7 (5—12)	11 (9—18)	11 (8—18)	**0.008**
Oligotrophic/atrophic nonunion	41 (93%)	11 (85%)	21 (84%)	0.400
Implant at presentation				0.119
Single lateral plate	39 (87%)	11 (85%)	17 (68%)	
Intramedullary nail	3 (6.7%)	0 (0%)	5 (20%)	
Nail-plate	0 (0%)	1 (7.7%)	0 (0%)	
Other	3 (6.7%)	1 (7.7%)	3 (12%)	
Mechanical implant failure	19 (43%)	7 (58%)	11 (46%)	0.646
Infected nonunion	6 (13%)	1 (7.7%)	2 (8.0%)	0.889
**Treatment characteristics**				
Retention of previous implants	10 (22%)	5 (38%)	0 (0%)	**0.003**
Fixation augmentation				** < 0.001**
Not augmented	45 (100%)	-	19 (76%)	
With endosteal plate	-	1 (7.7%)	6 (24%)	
With medial plate	-	5 (38%)	0 (0%)	
With IMN	-	7 (54%)	0 (0%)	
Autograft (RIA, ICBG or local callus)	23 (51%)	8 (62%)	4 (16%)	**0.005**
Bone graft substitute	17 (38%)	8 (62%)	2 (8.0%)	**0.001**
BMP	20 (44%)	4 (31%)	2 (8.0%)	**0.004**
DBM	13 (29%)	2 (15%)	0 (0%)	**0.004**
Cancellous chips	4 (8.9%)	2 (15%)	1 (4.0%)	0.411
Fibular strut^a^	2 (4.4%)	0 (0%)	0 (0%)	0.669

Bold indicates statistical significance (*p* < 0.05)

Missing values for: initial high-energy injury mechanism (n = 7, 8.4%), intra-articular fracture (n = 1, 1.2%), open fracture (n = 2, 2.4%), oligotrophic/atrophic nonunion (n = 1, 1.2%), mechanical implant failure (n = 1, 1.2%). Abbreviations: *IMN * intramedullary nail, *ICBG* iliac crest bone graft, *RIA* Reamer-Irrigator-Aspirator, *DBM* demineralized bone matrix

^a^One vascularized fibula and one fibula allograft

**Table 5:** Additional nonunion surgery rates by fixation construct

	All patients		Excluding patients with retention of previous implant	
Construct	Additional nonunion surgery	Rate (95%CI)	Additional nonunion surgery	Rate (95%CI)
Non-augmented lateral locking plate	12/45	27% (15%−42%)	7/35	20% (9.1%−37%)
Augmented lateral locking plate	1/13	7.7% (0.4%−38%)	0/8	0% (0%−40%)
Blade plate	1/25	4.0% (0.2%−22%)	1/25	4.0% (0.2%−22%)

Abbreviations: *CI * Confidence interval

## Discussion

Despite advances in implant design and surgical techniques, distal femur fractures continue to be associated with a high rate of reoperation for nonunion, ranging from 6 to 13% [1–5]. The mainstay treatment for distal femoral nonunion is surgical revision, however, there is limited evidence to guide treatment strategies, specifically regarding the choice for a fixation construct. In this study of 86 patients with a distal femoral nonunion who were treated with various surgical strategies, nonunion surgery was successful in 72 (84%) patients, while 14 (16%) patients failed treatment and required additional nonunion surgery. In unadjusted analysis, initial high-energy injuries, more previous surgeries, and treatment with retention of previous implants and BMP were associated with increased odds for additional nonunion surgery, whereas the use of blade plate constructs was associated with reduced odds. Definitive fixation with non-augmented lateral locking plate constructs showed higher failure rates (27%) when compared to augmented lateral locking plate constructs (7.7%) or blade plate constructs (4.0%).

To the authors’ best knowledge, the present series is the largest to investigate distal femoral nonunion repair cases. The rate of additional nonunion surgery of 16% is within range of what has been reported in previous series that used varying treatment strategies (0% to 20%) [6–10]. Gardner et al., described 31 patients that were treated with fixed-angle devices (including blade plates and lateral locked plates) with lag screw augmentation and ICBG or DBM [6]. Ninety-seven percent of patients healed after the index procedure with one patient being converted to a hinged knee replacement. Attum et al., reported on 10 patients treated with a lateral locking plate augmented with an intramedullary nail, RIA, and cancellous allograft in 6 patients [7]. One patient required incision and drainage for a deep infection, and all patients healed radiographically within the follow-up period. Poelmann et al., reported on 15 patients that were treated with lateral locked plating augmented with a medial PHILOS plate and ICBG [9]. Twelve (80%) patients healed after the index operation, with 3 patients requiring an additional bone grafting procedure. Landes et al., described 31 patients that were treated with a lateral locking plate (n = 30) or a blade plate (n = 1) and ICBG as needed [8]. Twenty-seven (87.1%) patients healed after the index procedure. Gavaskar et al., reported on 33 patients with “resistant” aseptic distal femoral nonunions, which were defined as nonunions that persisted for ≥ 12 months despite a minimum of two internal fixation procedures [10]. Patients were treated using a combination of a lateral and medial-based locking plate and autograft. Two (6%) patients had a persistent nonunion and were converted to endoprosthesis reconstruction or bone transport. The heterogeneity of treatment outcomes is likely due to varying nonunion, patient, and treatment characteristics. To guide treatment decisions, evidence on factors that are associated with such outcomes is helpful.

In the current study, initial high-energy injuries, more previous surgeries, and treatment with retention of previous implants and BMP were associated with increased odds for additional nonunion surgery. High-energy injuries typically result in greater damage to bone and soft tissues and may require more than one operative intervention (e.g., staged treatment with external fixation) [13]. The combined damage from the initial injury and repeated operative interventions may impair the local healing capacity. This is also supported by a previous study by Wiss et al. who identified that having three or more previous surgeries was a risk factor for treatment failure after adjusting for age, smoking, metabolic bone disease, open fracture, and history of infection in their cohort of 122 femoral nonunions [14]. The same group reported similar results in an analysis of 222 patients undergoing surgical treatment for tibial nonunions [15]. Combined, the findings that markers for the severity of the initial injury are associated with subsequent nonunion treatment outcomes is important for prognostication and patient education.

In the current study, definitive fixation with blade plate constructs reduced odds for nonunion when compared to non-augmented lateral locking plates, whilst augmented lateral locking plates did not. The absence of a significant association of augmented lateral locking plates is likely due to the small sample size. We therefore encourage readers to also consider the exploratory analysis of proportions of failures with 95% CIs when translating these results to practice. Specifically, non-augmented lateral locking plate failed in 27% of cases, which was considerably higher than the rate of 7.7% for augmented lateral locking plate constructs and 4.0% for blade plate constructs. The high failure rates of non-augmented lateral locking plate constructs may be attributed to cases treated with implant retention and bone grafting, suggesting that the trade-off between a lower-morbidity intervention and avoiding additional nonunion surgery should be carefully considered. When excluding patients treated with implant retention, failure rates of non-augmented lateral locking plates remained five times higher (20% vs. 0% and 4.0%), but the difference across groups did not reach statistical significance (p = 0.12). Failure rates were surrounded by wide confidence intervals, which relates to the relatively low event rates and sample size of groups. Nevertheless, these findings are consistent with current evidence. Biomechanical studies have demonstrated that dual-plate or nail-plate constructs are stronger compared to non-augmented lateral locking plates [16, 17]. Additionally, an increasing number of studies report promising clinical results when using these constructs for acute distal femur fractures [17, 18]. The low rates of additional nonunion surgery observed with the use of blade plates may be related to the implant or to the technique of applying the blade plate in compression while preserving the vascularity of the bone. Clinical studies also observed low failure rates with the use of blade plates. In a retrospective study, Vallier et al. identified a trend towards a lower nonunion rate (3.4% vs. 16%, p = 0.11) using 95-degree angled blade plates when compared to lateral locking plates for acute distal femur fractures [19]. In a subsequent randomized clinical trial, 0/34 (0%) patients treated with an blade plate developed nonunion compared to 3/45 (7%) treated with a lateral locking plate (p = 0.06) [20]. Combined, previous data and the current study results support the use of augmented lateral locking plate or blade plate constructs in the treatment of distal femoral nonunions.

The comparison of fixation constructs should be interpreted in the context of differences in nonunion and treatment characteristics across groups. Patients treated with non-augmented lateral locking plates had a relatively shorter nonunion duration, fewer previous nonunion surgeries, more often an initial intra-articular fracture, and were more often treated with implant retention. Fixation constructs also represented different treatment approaches. Both augmented and non-augmented lateral locking plate constructs were more often combined with autograft or bone graft substitutes. Despite these differences, the high failure rate of non-augmented lateral locking plates suggests that these constructs should be avoided in distal femoral nonunion repair.

There was a significant and strong association with use of BMP and additional nonunion surgery, although this finding is likely confounded by the construct type as the use of BMPs was highest in the non-augmented lateral locking plate constructs. There may also be indication bias arising from the use of BMPs for nonunions that were considered to be more severe. Regardless, the use of BMPs remains controversial and there is no definitive evidence that they increase the chances of healing [21].

This study has limitations. First, the retrospective nature of the study may have introduced selection bias and there were differences in nonunion and patient characteristics between fixation construct groups. In addition, there was no pre-defined treatment algorithm, and it was not possible to retrospectively quantify specific indications for treatment decisions and additional outcomes, such as final ambulatory status or range of motion. However, the study included a range of explanatory variables to provide insight into the characteristics across treatment groups. Unfortunately, performing multivariable logistic regression to mitigate these limitations was not possible due to the low event rate (n = 14). This would introduce risk for overfitting and therefore would limit any clinical utility. In addition, final radiographic healing was not reported. Litrenta et al. demonstrated that the (modified) Radiographic Union Scale for Tibia (RUST) had moderate interobserver reliability for the interpretation of healing of distal femur fractures, but reliability was negatively affected by the presence of extramedullary implants obscuring the cortex [22]. Also, the use of compression for many patients may not result in healing with callus formation. These factors may introduce significant bias in radiographic assessment of healing and the primary outcome was therefore determined as additional nonunion surgery. Second, the retrospective nature did not allow for a standardized preoperative and postoperative assessment. Specifically, measures for metabolic and endocrine abnormalities that may influence healing (e.g., calcium or vitamin D) were only scarcely obtained, and intraoperative culture sample protocols were not standardized, which limits the diagnosis of occult infection [23–25]. It should be emphasized that the study included a heterogeneous mix of nonunion types (e.g., periprosthetic vs. native) and that there is an inherent relationship between patient, nonunion, and treatment characteristics that may not be captured with the analyses performed in the present study. Given the complexity of nonunion treatment and the relationship between the number of surgeries and patient-reported outcomes, studies with larger sample sizes that allow stratification in more homogeneous samples and multivariable analysis are needed [24].

In conclusion, in this cohort of 86 distal femoral nonunions treated with a variety of surgical strategies, approximately 1 in 6 patients required additional nonunion surgery. In unadjusted analyses, initial high-energy injuries and more prior surgeries were associated with increased odds for additional nonunion surgery, suggesting that the severity of the initial injury is associated with subsequent nonunion treatment outcomes. The current study findings suggest that distal femoral nonunion repair should be based on revision fixation using augmented lateral locking plate (dual-plate or nail-plate) or blade plate constructs. However, these findings are based on unadjusted comparisons. Larger studies with sufficient power to correct or stratify for confounding are needed to further define optimal treatment.

## Data Availability

No datasets were generated or analysed during the current study.
